# Age at Disease Onset Associates With Oxidative Stress, Neuroinflammation, and Impaired Synaptic Plasticity in Relapsing-Remitting Multiple Sclerosis

**DOI:** 10.3389/fnagi.2021.694651

**Published:** 2021-09-10

**Authors:** Mario Stampanoni Bassi, Luana Gilio, Ennio Iezzi, Alessandro Moscatelli, Tatjana Pekmezovic, Jelena Drulovic, Roberto Furlan, Annamaria Finardi, Georgia Mandolesi, Alessandra Musella, Giovanni Galifi, Roberta Fantozzi, Paolo Bellantonio, Marianna Storto, Diego Centonze, Fabio Buttari

**Affiliations:** ^1^Unit of Neurology and Neurorehabilitation, IRCCS Neuromed, Pozzilli, Italy; ^2^Department of Systems Medicine, Tor Vergata University, Rome, Italy; ^3^Laboratory of Neuromotor Physiology, IRCCS Fondazione Santa Lucia, Rome, Italy; ^4^Institute of Epidemiology, Faculty of Medicine, University of Belgrade, Belgrade, Serbia; ^5^Clinic of Neurology, Clinical Center of Serbia, Belgrade, Serbia; ^6^Clinical Neuroimmunology Unit, Institute of Experimental Neurology, Division of Neuroscience, San Raffaele Scientific Institute, Milan, Italy; ^7^Synaptic Immunopathology Lab, IRCCS San Raffaele Pisana, Rome, Italy; ^8^Department of Human Sciences and Quality of Life Promotion, San Raffaele University, Rome, Italy

**Keywords:** aging, relapsing remitting multiple sclerosis, progression of disability independent of disease activity, synaptic plasticity, neuroinflammation, oxidative stress

## Abstract

Age at onset is the main risk factor for disease progression in patients with relapsing-remitting multiple sclerosis (RR-MS). In this cross-sectional study, we explored whether older age is associated with specific disease features involved in the progression independent of relapse activity (PIRA). In 266 patients with RR-MS, the associations between age at onset, clinical characteristics, cerebrospinal fluid (CSF) levels of lactate, and that of several inflammatory molecules were analyzed. The long-term potentiation (LTP)-like plasticity was studied using transcranial magnetic stimulation (TMS). Older age was associated with a reduced prevalence of both clinical and radiological focal inflammatory disease activity. Older patients showed also increased CSF levels of lactate and that of the pro-inflammatory molecules monocyte chemoattractant protein 1 (MCP-1)/CCL2, macrophage inflammatory protein 1-alpha (MIP-1α)/CCL3, and interleukin (IL)-8. Finally, TMS evidenced a negative correlation between age and LTP-like plasticity. In newly diagnosed RR-MS, older age at onset is associated with reduced acute disease activity, increased oxidative stress, enhanced central inflammation, and altered synaptic plasticity. Independently of their age, patients with multiple sclerosis (MS) showing similar clinical, immunological, and neurophysiological characteristics may represent ideal candidates for early treatments effective against PIRA.

## Introduction

Multiple sclerosis (MS) is an inflammatory immune-mediated disease of the central nervous system (CNS) with a highly variable disease course. While the relapsing-remitting (RR) course is better described in terms of episodical aggression of the brain by peripheral autoreactive T and B lymphocytes, factors involved in the progressive phenotypes [primary progressive (PP) and secondary progressive (SP)] are believed to take place in the CNS (Faissner et al., 2019). Increased mitochondrial dysfunction, oxidative stress, and exacerbated intrathecal inflammation may promote axonal damage and neurodegeneration, playing crucial roles in the progression independent of relapse activity (PIRA) (Faissner et al., 2019). In addition, impaired synaptic plasticity, and particularly absent long-term potentiation (LTP), may critically contribute to progressive clinical deterioration, weakening the ability to compensate for ongoing brain damage (Mori et al., 2013).

Drugs approved as first-line agents in relapsing-remitting multiple sclerosis (RR-MS) significantly reduce relapse rate and disability worsening related to relapse activity, showing less or no effect in progressive MS phenotypes. The introduction of therapies effective in progressive MS, such as siponimod and ocrelizumab, stimulated the interest in understanding the determinants of progression.

Age at disease onset is one of the main predictors of early transition to the progressive phenotype in patients with RR-MS (Tutuncu et al., 2013) incorporated in accepted indexes of SP conversion risk at diagnosis [i.e., Bayesian risk estimate for MS at onset (BREMSO)] (Bergamaschi et al., 2015). What distinguishes newly diagnosed younger and older patients with MS is largely unknown. Thus, exploring whether older age is associated with specific disease features involved in PIRA could be of paramount importance to tailor therapeutic choices even in young patients. In this study, we investigated in a group of patients with RR-MS associations between age at onset, clinical characteristics, levels of lactate, and a large set of inflammatory molecules in the cerebrospinal fluid (CSF). In addition, association with synaptic plasticity was also explored.

## Methods

### Patients With MS, Clinical Evaluation, and MRI

A group of 266 patients with RR-MS admitted to the neurology clinic of the Neuromed Hospital (Pozzilli, IS) between 2017 and 2020 participated in this cross-sectional study. RR-MS diagnosis was based on the clinical, laboratory, and MRI parameters (Thompson et al., 2018). This study was approved by the Ethics Committee of the Neuromed Hospital. All patients gave written informed consent.

At the time of MS diagnosis, the following clinical variables were evaluated. Disease duration was calculated as the time interval between the first episode of focal neurological dysfunction suggestive of MS and the confirmed diagnosis. Age at onset was calculated by subtracting disease duration from age at the time of diagnosis. Clinical disability was evaluated with the Expanded Disability Status Scale (EDSS) (Kurtzke, 1983). BREMSO was calculated (Bergamaschi et al., 2015). The clinical disease activity was defined as the presence of symptoms of focal damage at the time of diagnosis.

The MRI examination consisted of a 1.5- or 3.0-Tesla scan, including dual-echo proton density sequences, FLAIR, T1-weighted spin-echo (SE), T2-weighted fast SE, and contrast-enhanced T1-weighted SE after intravenous gadolinium (Gd) infusion (0.2 ml/kg). The radiological disease activity was defined as the presence of a gadolinium-enhancing (Gd+) lesion at the brain and a spine MRI scan performed at the time of diagnosis.

Cerebrospinal fluid sample collection and transcranial magnetic stimulation (TMS) were performed before the administration of corticosteroids or immunoactive therapies.

### CSF Collection and Analysis

The CSF was collected by lumbar puncture (LP). Of note, 2 ml was used for the biochemical analyses, including total cell count and lactate levels. The presence of oligoclonal bands (OCB) was assessed. After LP, CSF was centrifuged and stored at −80°C. Pro-inflammatory and anti-inflammatory molecules were analyzed using a Bio-Plex multiplex cytokine assay (Bio-Rad Laboratories, Hercules, CA, USA). The CSF molecules examined were included as follows: interleukin (IL)-1β, IL-2, IL-4, IL-5, IL-6, IL-7, IL-8, IL-9, IL-10, IL-12, IL-13, IL-15, IL-17, IL-1ra, granulocyte colony-stimulating factor (G-CSF), granulocyte-macrophage colony-stimulating factor (GM-CSF), IP-10, interferon (IFN)-γ, tumor necrosis factor (TNF), monocyte chemoattractant protein 1 (MCP-1)/CCL2, regulated on activation, normal T cell expressed and secreted (RANTES), macrophage inflammatory protein 1-alpha (MIP-1α)/CCL3. All samples were analyzed in triplicate. Concentrations were expressed as picograms/milliliters.

### Transcranial Magnetic Stimulation

Motor evoked potentials (MEPs) were elicited using a figure-of-eight coil, with an external loop diameter of 70 mm, connected to a Magstim 200^2^ magnetic stimulator (The Magstim Company, Whitland, Wales, UK). The coil was positioned in the optimal position (hot spot) for eliciting MEPs in the right first dorsal interosseus (FDI) muscle. Surface electromyographic responses were amplified using a Digitimer D360 amplifier (Digitimer, Welwyn Garden City, Hertfordshire, UK). Filters were set at 20 Hz and 2 kHz with a sampling rate of 5 kHz. Traces were stored on a computer with SIGNAL software (Cambridge Electronic Devices, Cambridge, UK). Intermittent theta burst stimulation (iTBS) was applied to the right FDI motor hot spot using a Magstim Rapid^2^ stimulator, with a stimulation intensity of 80% of the active motor threshold (AMT) (Huang et al., 2005). The AMT was calculated as the minimum stimulus intensity for evoking MEPs of approximately 200 μV in 50% of 10 consecutive trials from slightly contracted FDI muscle. Twenty MEPs were recorded from the relaxed FDI muscle prior to iTBS, with intensity set to elicit stable MEPs of 0.5–1 mV amplitude. Using the same stimulation intensity, 20 MEPs were collected 5, 15, and 30 min after iTBS. At each time point, MEPs were averaged and normalized to the mean baseline amplitude.

### Statistical Analysis

The normality test was performed using the Kolmogorov–Smirnov test. The data were shown as median [interquartile range (IQR)]. Categorical variables were presented as counts (*n*) and frequency (%). Association between categorical variables was examined by applying the χ^2^ test. Differences in continuous variables among two groups were evaluated by using either the parametric *t*-test or the nonparametric Mann–Whitney U test. A *p* ≤ 0.05 was considered statistically significant. Spearman's nonparametric correlation and partial correlation were used to test possible associations between variables that were not normally distributed. The Benjamini–Hochberg (B–H) procedure was used to control the false discovery rate and the Type I errors (false positives). To explore associations between age and different variables, after adjustment for possible confounding factors, linear or logistic regression models were used (for continuous and dichotomous variables, respectively). The Box plot was used to depict statistically significant differences between groups. Multiple linear regression models were used to evaluate the effect of age, sex, and groups (patients with MS vs. controls) on the TMS results.

All analyses were performed using IBM SPSS Statistics for Windows (IBM Corp., Armonk, NY, USA).

The missing data were as follows: disease duration 1/266 (0.4%); OCB 5/266 (1.9%); clinical activity 3/266 (1.1%); radiological activity 14/266 (5.2%); BREMSO 14/266 (5.2%); CSF lactate 11/266 (4.1%); and CSF cytokines 20/266 (7.5%).

## Results

### Age and Clinical Characteristics

The clinical characteristics of patients with RR-MS are shown in Table 1.

**Table 1:** Demographic and clinical characteristics of patients with relapsing-remitting multiple sclerosis (RR-MS).

Patients	*N* (%)	266
Sex (F)	*N* (%)	183/266 (68.8)
Age at onset (years)	Median (IQR)	32.93 (25.5–42.5)
Disease duration (months)	Median (IQR)	4.97 (1.27–25.08)
Unique CSF OCB, present	*N* (%)	190/261 (72.8)
EDSS	Median (IQR)	1.5 (1–2.5)
Clinical disease activity, yes	*N* (%)	94/263 (35.7)
Radiological disease activity, yes	*N* (%)	108/252 (42.9)
BREMS at onset	Median (IQR)	0.02 (−0.55 to 0.58)

*BREMSO, Bayesian risk estimate for MS at onset; CSF, cerebrospinal fluid; EDSS, Expanded Disability Status Scale; MS, multiple sclerosis; OCB, oligoclonal bands; RR, relapsing-remitting*.

**Figure 1:**
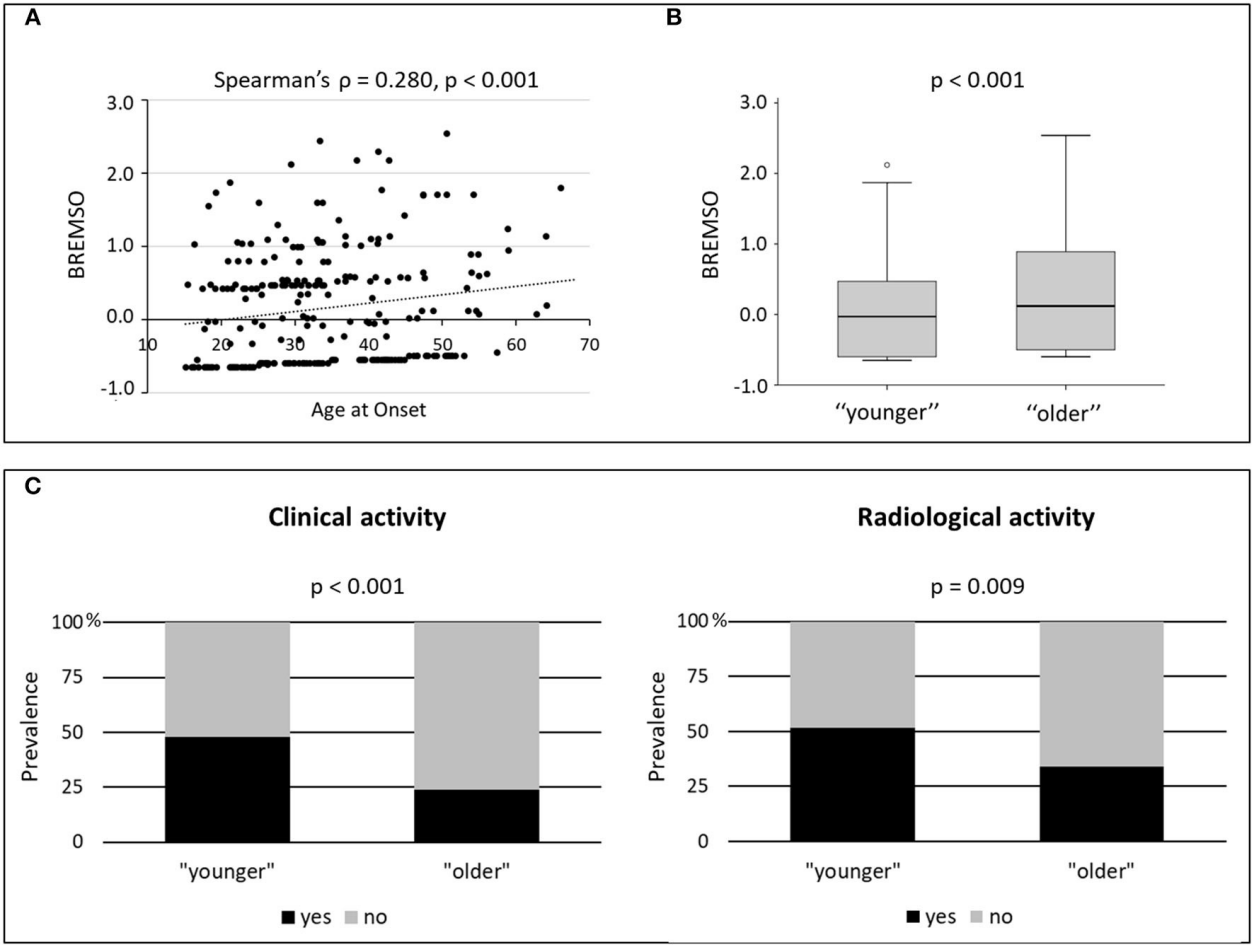
Age and clinical characteristics. **(A)** Correlation between age at onset and BREMSO; Spearman's ρ and *p*-value are shown. **(B)** BREMSO in “younger” and “older” patients with multiple sclerosis (MS); the B–H adjusted *p*-value of the Mann–Whitney *U* test is shown. **(C)** Prevalence of clinical and radiological disease activity in “younger” and “older” patients with MS; the B–H adjusted *p*-values of the χ^2^ test are shown. B–H, Benjamini–Hochberg; BREMSO, Bayesian risk estimate for MS at the onset.

Age at onset showed significant positive correlations with both disease duration (Spearman's ρ = 0.183, *p* = 0.003) and BREMSO (Spearman's ρ = 0.280, *p* < 0.001) (Figure 1A). The correlation between age of onset and BREMSO was significant, also correcting for the effect of disease duration by means of partial correlation (Spearman's ρ = 0.249, *p* < 0.001). Conversely, no significant correlation was observed between age at onset and EDSS (*p* = 0.341), and no significant associations emerged between age at onset and sex (*p* = 0.915) or the presence of OCB (*p* = 0.125).

To better explain the impact of age at onset on clinical characteristics, patients with MS were divided, according to the median value (32.93 years), into two groups: “younger” [133 patients: median (IQR) = 25.5 (21.68–29.39)], and “older” [133 patients: median (IQR) = 42.49 (37.82–49.29)] (Table 2). Significant differences were found between the two groups in disease duration (*p* = 0.003; B–H adjusted *p* = 0.007) and BREMSO (*p* < 0.001; B–H adjusted *p* < 0.001) (Figure 1B). Linear regression showed that the age group was significantly associated with BREMSO after adjusting for all other clinical variables (i.e., sex, disease duration, EDSS, OCB presence, and clinical and radiological activity) (beta coefficient = 0.136, 95% CI 0.066–0.360, *p* = 0.005).

**Table 2:** Clinical characteristics of patients with RR-MS according to the age group.

		**“Younger”**	**“Older”**	**B–H adjusted *p***
Patients	*N*	133	133	
Age at onset, years	Median (IQR)	25.5 (21.68–29.39)	42.49 (37.82–49.29)	–
Sex, F	*N* (%)	89 (66.9)	94 (70.7)	0.508^*^
Disease duration, months	Median (IQR)	3 (0.97–16.95)	10.7 (1.96–36.25)	0.007^#^
Unique CSF OCB, present	*N* (%)	98/130 (75.4)	92/131 (70.2)	0.407^*^
EDSS	Median (IQR)	1.5 (1–2)	2 (1–2.5)	0.281^#^
Clinical disease activity, yes	*N* (%)	63/132 (47.7)	31/131 (23.7)	<0.001^*^
Radiological disease activity, yes	*N* (%)	66/128 (51.6)	42/124 (33.9)	0.009^*^
BREMS at onset	Median (IQR)	−0.03 (−0.6 to 0.47)	0.12 (−0.5 to 0.89)	<0.001^#^

* *p-value of the χ^2^ test;*

# *p-value of the Mann–Whitney U test*.

*B–H, Benjamini–Hochberg; BREMSO, Bayesian risk estimate for MS at onset; CSF, cerebrospinal fluid; EDSS, Expanded Disability Status Scale; MS, multiple sclerosis; OCB, oligoclonal bands; RR, relapsing-remitting*.

Significant differences were found between the two age groups, also considering the prevalence of clinical and radiological acute focal disease activity at the time of diagnosis. Reduced prevalence of both clinical (*p* < 0.001; B–H adjusted *p* < 0.001) and radiological (*p* = 0.005; B–H adjusted *p* = 0.009) disease activity was observed in the “older” RR-MS group (Figure 1C). Logistic regressions for the binary data confirmed the significant associations between age group and both clinical [odds ratio (OR) = 3.255, 95% CI 1.839–5.763, *p* < 0.001] and radiological disease activity [OR = 2.118, 95% CI 1.246–3.598, *p* = 0.006], controlling for sex, disease duration, EDSS, and OCB presence. The results suggest that older age at onset is characterized by the reduced prevalence of both clinical and radiological disease activity and is associated with a higher risk of secondary progression as shown by higher BREMSO.

As a cutoff of 40 years of age at onset is commonly used in the literature to differentiate late-onset RR-MS (LORRMS) from young-onset RR-MS (YORRMS) (D'Amico et al., 2018), patients were further analyzed using such definition (Table 3). Significant differences were found between YORRMS and LORRMS in BREMSO (*p* = 0.011; B–H adjusted *p* = 0.038), and in clinical disease activity (*p* = 0.002; B–H adjusted *p* = 0.014). Logistic regression confirmed a significant association between age group (YORRMS vs. LORRMS) and clinical activity (OR = 2.839, 95% CI 1.511–5.334, *p* = 0.001). Conversely, linear regression showed that the association between age group (YORRMS vs. LORRMS) and BREMSO was not significant after adjusting for all other clinical variables (i.e., sex, disease duration, EDSS, OCB presence, and clinical and radiological activity) (*p* = 0.33).

**Table 3:** Clinical characteristics of young-onset and late-onset patients with RR-MS.

		**YORRMS**	**LORRMS**	**B–H adjusted *p***
Patients	*N*	177	89	
Age of onset (years)	Median (IQR)	28.16 (23.13–32.93)	47.37 (42.46–51.53)	–
Sex, F	*N* (%)	117 (66.1)	66 (74.2)	0.211^*^
Disease duration, months	Median (IQR)	3.71 (1.07–21.17)	11.4 (1.7–37.6)	0.11^#^
Unique CSF OCB, present	*N* (%)	128/172 (74.4)	62/89 (69.7)	0.413^*^
EDSS	Median (IQR)	1.5 (1–2)	2 (1–2.5)	0.11^#^
Clinical disease activity, yes	*N* (%)	74/176 (42)	20/87 (23)	0.014^*^
Radiological disease activity, yes	*N* (%)	79/170 (46.5)	29/82 (35.4)	0.133^*^
BREMS at onset	Median (IQR)	0.02 (−0.6 to 0.53)	0.07 (−0.5 to 0.76)	0.038^#^

* *p-value of the χ^2^ test;*

# *p-value of the Mann–Whitney U test*.

*B–H, Benjamini–Hochberg; BREMSO, Bayesian risk estimate for MS at onset; CSF, cerebrospinal fluid; EDSS, Expanded Disability Status Scale; YORRMS, young-onset relapsing-remitting multiple sclerosis; LORRMS, late-onset relapsing-remitting multiple sclerosis; MS, multiple sclerosis; OCB, oligoclonal bands; RR, relapsing-remitting*.

### Age and CSF Lactate

A significant correlation was found between age at onset and CSF lactate (Spearman's ρ = 0.281, *p* < 0.001, B–H adjusted *p* < 0.01, *N* = 255), also after correcting for the effect of disease duration (partial Spearman's ρ = 0.250, *p* < 0.001).

**Figure 2:**
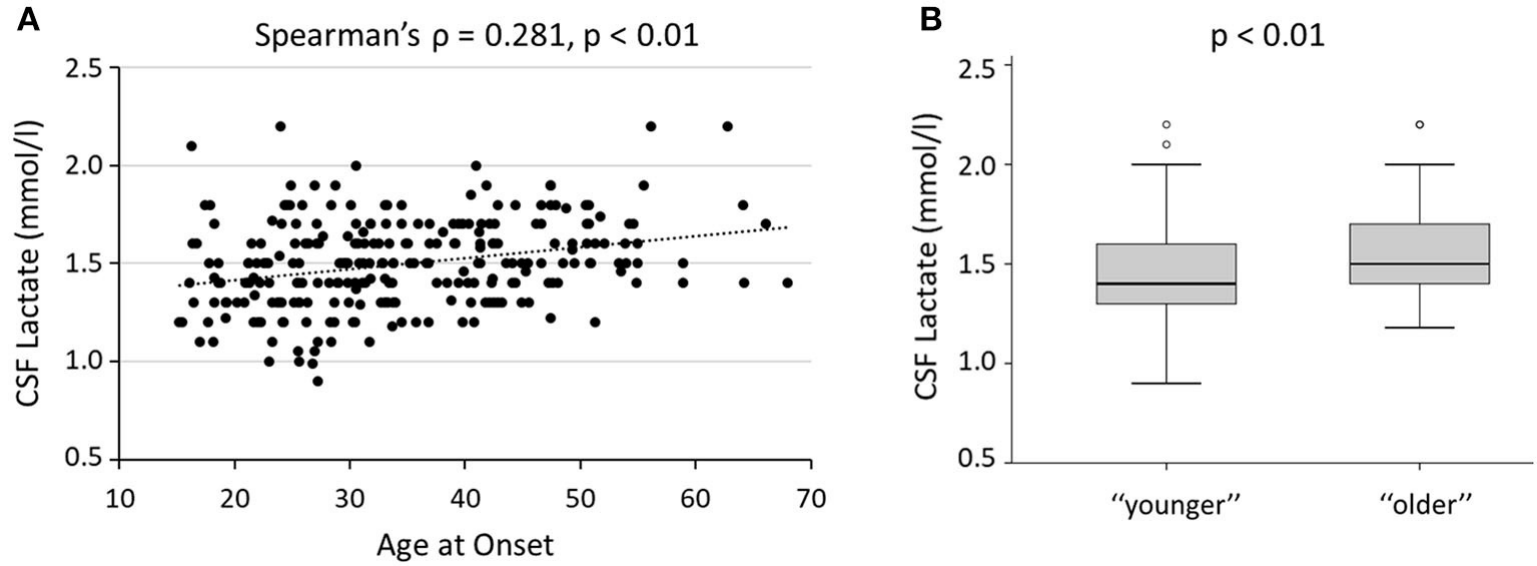
Age and CSF lactate. **(A)** Correlation between age at onset and CSF lactate levels; Spearman's ρ and B–H adjusted *p*-value are shown. **(B)** CSF lactate in “younger” and “older” patients with MS; the B–H adjusted *p*-value of the Mann–Whitney *U* test is shown. B–H, Benjamini–Hochberg; CSF, cerebrospinal fluid.

In addition, CSF lactate levels were significantly increased in “older” patients with MS [“younger” median (IQR) = 1.4 (1.3–1.6); “older” median (IQR) = 1.5 (1.4–1.7); *p* < 0.001; B–H adjusted *p* < 0.01] (Figure 2). Linear regression showed that this difference was significant, also controlling for all other clinical variables (beta coefficient = 0.157, 95% CI 0.011–0.131, *p* = 0.021).

Significant differences were confirmed, also comparing CSF lactate levels in patients with YORRMS and LORRMS [YORRMS median (IQR) = 1.43 (1.3–1.6); LORRMS median (IQR) = 1.57 (1.4–1.7); *p* < 0.001, B–H adjusted *p* < 0.01]. Linear regression confirmed a significant association between age group (YORRMS vs. LORRMS) and CSF lactate levels, also controlling for all other clinical variables (beta coefficient = 0.197, 95% CI 0.032–0.157, *p* = 0.003).

### Age and CSF Inflammation

**Figure 3:**
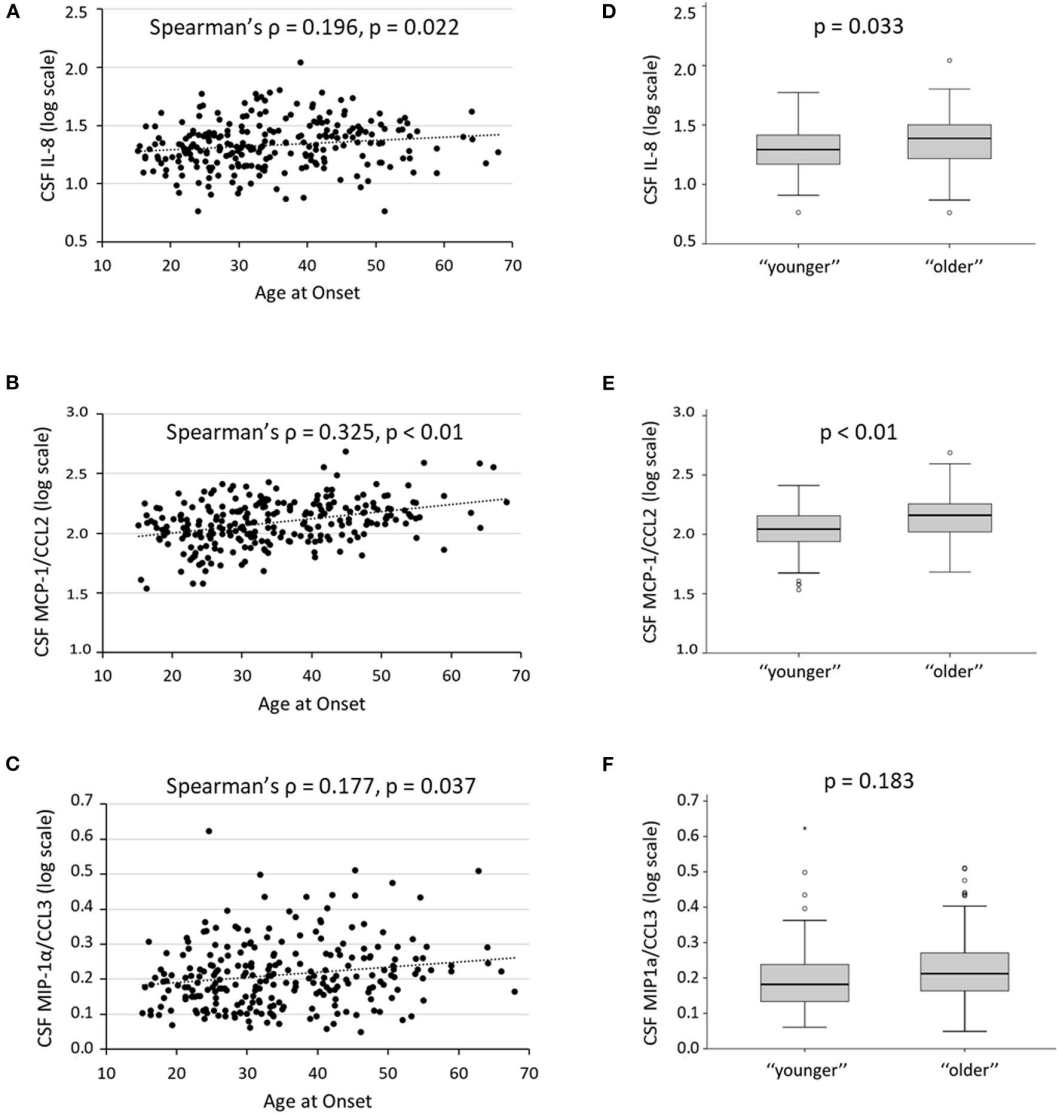
Age and CSF inflammation. **(A–C)** Correlations between age at onset and CSF levels of IL-8, MCP-1, and MIP-1α; Spearman's ρ and B–H adjusted *p*-values are shown. **(D–F)** CSF levels of IL-8, MCP-1, and MIP-1α, in “younger” and “older” patients with MS; the B–H adjusted *p*-values of the Mann–Whitney U test are shown. To obtain a better graphical representation, CSF cytokines concentrations are shown on a logarithmic scale. B–H, Benjamini–Hochberg; CSF, cerebrospinal fluid; IL, interleukin; MCP-1, monocyte chemoattractant protein 1; MIP-1α, macrophage inflammatory protein 1-alpha.

Significant correlations were found between age at onset and the inflammatory molecules IL-8 (Spearman's ρ = 0.196, *p* = 0.002, B–H adjusted *p* = 0.022), MCP-1/CCL2 (Spearman's ρ = 0.325, *p* < 0.001, B–H adjusted *p* < 0.01), and MIP-1α/CCL3 (Spearman's ρ = 0.177, *p* = 0.005, B–H adjusted *p* = 0.037) (Figures 3A–C). These correlations were significant, also controlling for the effect of disease duration (IL-8, partial Spearman's ρ = 0.193, *p* = 0.002; MCP-1/CCL2, partial Spearman's ρ = 0.320, *p* < 0.001; MIP-1α/CCL3, partial Spearman's ρ = 0.162, *p* = 0.011). No significant correlations were found between age and the other CSF molecules analyzed (Supplementary Table).

Comparing “younger” and “older” MS groups, significant differences were found in IL-8 [“younger” median (IQR) = 18.62 (13.67–25.02); “older” median (IQR) = 23.41 (15.47–30.78); *p* = 0.003, B–H adjusted *p* = 0.033] and MCP-1/CCL2 [“younger” median (IQR) = 109.71 (85.79–143.43); “older” median (IQR) = 143.72 (102.55–179.89); *p* < 0.001, B–H adjusted *p* < 0.01] CSF levels. Conversely, the difference in MIP-1α/CCL3 CSF levels was not significant after correction for multiple comparisons [“younger” median (IQR) = 0.52 (0.35–0.73); “older” median (IQR) = 0.63 (0.46–0.87); *p* = 0.025, B–H adjusted *p* = 0.183] (Figures 3D–F). Linear regression confirmed a significant association with IL-8 (beta coefficient = 0.194, 95% CI 1.609–8.212, *p* = 0.004) and MCP-1/CCL2 (beta coefficient = 0.256, 95% CI 15.351–48.016, *p* < 0.001), also adjusting for all other clinical variables.

Finally, when comparing CSF cytokines in patients with YORRMS and LORRMS, significant differences were found in IL-8 [YORRMS median (IQR) = 19 (13.31–25.57); LORRMS median (IQR) = 24.45 (16.22–30.95); *p* = 0.004, B–H adjusted *p* = 0.044] and MCP-1/CCL2 [YORRMS median (IQR) = 111.24 (87.65–150.60); LORRMS median (IQR) = 147.04 (117.64–181.25); *p* < 0.001, B–H adjusted *p* < 0.01]; conversely, association with MIP-1α/CCL3 [YORRMS median (IQR) = 0.54 (0.35–0.75); LORRMS median (IQR) = 0.67 (0.48–0.92); *p* = 0.012, B–H adjusted *p* = 0.088] was not significant after controlling for multiple comparisons.

To further exclude that different clinical and radiological activity in the two age groups could affect CSF cytokine levels, we performed additional analyses by excluding patients with clinical or radiological activity at the time of CSF collection. When comparing patients with stable YORRMS and LORRMS, significant differences were found only in MCP-1/CCL2 CSF levels [YORRMS median (IQR) = 109.7 (88.98–149.91); LORRMS median (IQR) = 146.57 (117.68–179.37); *p* < 0.001, B–H adjusted *p* < 0.01]. Associations emerged also with IL-5 [YORRMS median (IQR) = 0.03 (0–1.74); LORRMS median (IQR) = 1 (0.3–2.37); *p* = 0.035, B–H adjusted *p* = 0.21], IL-8 [YORRMS median (IQR) = 17.59 (12.88–24.41); LORRMS median (IQR) = 24.43 (15.45–28.22); *p* = 0.04, B–H adjusted *p* = 0.21], and IL-12 [YORRMS median (IQR) = 0.01 (0–1.07); LORRMS median (IQR) = 0.85 (0.03–1.22); *p* = 0.017, B–H adjusted *p* = 0.18], although not significant after correction for multiple comparisons. Linear regression confirmed a significant association between MCP-1/CCL2 CSF levels and age group (YORRMS vs. LORRMS), also adjusting for other clinical characteristics (i.e., sex, disease duration, EDSS, and OCB presence) (beta coefficient = 0.343, 95% CI 17.055–63.625, *p* = 0.001).

### Age and TMS-Induced Synaptic Plasticity

Transcranial magnetic stimulation was performed at the time of diagnosis in 40 clinical and radiological patients with stable RR-MS unaffected in the right upper limb [sex, F: *N* (%) = 28 (70); age, median (IQR) = 31.69 (24.34–43.01); EDSS median (IQR) = 1 (1–2); OCB presence: *N* (%) = 23 (57.5)]. TMS was well tolerated, and no adverse events were reported.

A significant negative correlation emerged between age and the magnitude of the LTP-like effect induced by iTBS (post 5: Spearman's ρ = −0.407, *p* = 0.009; post 15: Spearman's ρ = −0.326, *p* = 0.04; post 30: Spearman's ρ = −0.379, *p* = 0.016). Linear regression showed a significant association between age at onset and iTBS effect adjusting for potential confounders (i.e., sex, disease duration, EDSS, and OCB presence) (post 5: beta coefficient = −0.464, 95% CI −0.011 to −0.002, *p* = 0.008; post 15: beta coefficient = −0.519, 95% CI −0.012 to −0.003, *p* = 0.002; post 30: beta coefficient = −0.469, 95% CI −0.013 to −0.002, *p* = 0.008).

**Figure 4:**
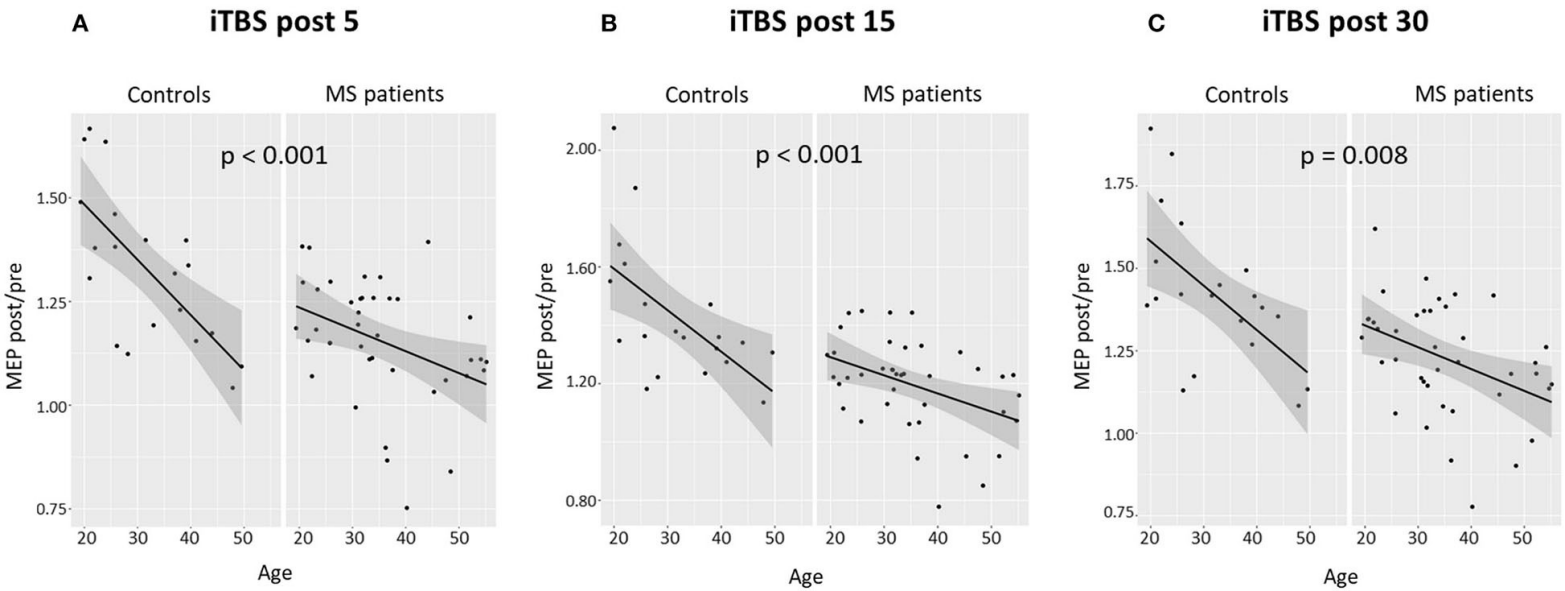
Age and LTP-like synaptic plasticity in patients with MS and control subjects. Correlation between age and iTBS-induced effects in patients with MS and controls at different time points: **(A)** 5 min, **(B)** 15 min, and **(C)** 30 min. The data are expressed as mean MEP amplitudes (MEP post) normalized to the mean baseline MEP (MEP pre). Multiple linear regression models evidenced a significant effect of age at all time points both in patients with MS and in the control group. B–H adjusted *p*-values are shown. B–H, Benjamini–Hochberg; LTP, long-term potentiation; iTBS, intermittent theta burst stimulation; MEP, motor evoked potential.

Results obtained in patients with MS were compared with a group of 20 healthy control subjects [sex, F: *N* (%) = 14 (70); age, median (IQR) = 29.85 (23.5–39.22)]. Multiple linear regression models were used to evaluate the impact of age, sex, and group (patients with MS vs. controls) on iTBS-induced LTP-like plasticity. As shown in Figure 4, both in patients with MS and in the control group, a significant effect of age was evidenced at all time points (post 5, *p* < 0.001; post 15, *p* < 0.001; post 30, *p* = 0.008). Furthermore, a significant main effect of the group was evidenced, with patients with MS showing reduced LTP-like effect induced by the iTBS protocol at all time points compared with healthy controls (post 5, *p* = 0.008; post 15, *p* = 0.001; post 30, *p* = 0.008).

## Discussion

Early identification of patients at risk for progression is important for developing effective therapeutic strategies. This would also allow the timely initiation of specific treatments. To understand the mechanisms involved in MS progression, we examined a category of patients at risk for secondary progression, namely, patients with RR-MS with older age at onset. We explored whether these patients could display specific disease characteristics resembling those of progressive patients. As expected, older patients with RR-MS had higher BREMSO scores, confirming an increased risk of progression to SP-MS. Older age at onset was also independently associated with the reduced prevalence of acute focal disease activity, likely contributing to delay MS diagnosis. The absence of acute inflammatory events and the development of a chronic unremitting inflammation signal the shift toward the progressive disease stages of MS (Filippi et al., 2020). Reduced disease reactivations may therefore represent a shared trait between older patients with RR-MS and progressive patients.

Specific pathogenetic mechanisms promote axonal damage and neurodegeneration in progressive MS, including compartmentalized inflammation, oxidative stress, and mitochondrial damage (Faissner et al., 2019; Monaco et al., 2020). Cellular senescence is characterized by increased release of cytokines, metalloproteinases, and reactive oxygen species (ROS) by innate and adaptive immune cells, astrocytes, and neurons (Hou et al., 2019; Papadopoulos et al., 2020). Increased release of ROS and mitochondrial dysfunction are involved in axonal and neuronal damage (Su et al., 2013), and these changes may promote neurodegeneration, representing an important link between aging and MS progression (Musella et al., 2018). We evidenced a positive correlation between age at onset and CSF lactate levels in patients with RR-MS. Although other biomarkers of oxidative stress have not been examined, the CSF lactate levels have been previously proposed as a biomarker of mitochondrial dysfunction and have been associated with higher EDSS and CSF neurofilament levels in RR-MS (Albanese et al., 2016). Our results likely reflect increased oxidative stress and mitochondrial dysfunction in older patients with RR-MS, possibly representing an independent source of clinical deterioration in these patients.

The development of chronic intrathecal inflammation represents another important factor involved in MS progression. We found that older patients with RR-MS display increased levels of specific pro-inflammatory cytokines, such as MCP-1/CCL2, IL-8, and MIP-1α/CCL3. These molecules are the important pro-inflammatory mediators involved in the pathogenesis of experimental autoimmune encephalomyelitis (EAE) and MS. Increased CSF levels of IL-8 have been previously reported in patients with MS compared to controls (Matejčíková et al., 2017) and have been associated with worse MS disease course (Stampanoni Bassi et al., 2018). MIP-1α/CCL3 is released by T and B lymphocytes, macrophages, neutrophils, and microglia. This pro-inflammatory molecule regulates cell differentiation, chemotaxis, and activation, promoting the release of various inflammatory mediators (Maurer and von Stebut, 2004). Increased MIP-1α/CCL3 expression has been reported in T lymphocytes of patients with MS and astrocytes and macrophages within the plaque (Balashov et al., 1999). Notably, in patients with RR-MS treated with IFN-β, MIP1α/CCL3 CSF undetectability predicted the absence of clinical/radiological relapses and disability progression 1 year after diagnosis (Stampanoni Bassi et al., 2020). In this study, a strong association between MCP-1/CCL2 and age was evidenced. MCP-1/CCL2 binds to the CCR2 receptor, which is expressed by a wide set of immune cells and regulates the activation and migration of macrophages and microglia (Simpson et al., 1998; Chen et al., 2003). Increased infiltration and activation of macrophages and microglial cells has been evidenced in the brain of transgenic mice overexpressing CCL2 (Mildner et al., 2009). Although the activity of this molecule in MS pathogenesis is not fully defined, the enhanced expression of CCR2 has been reported in T cells from patients with SP-MS (Sørensen and Sellebjerg, 2001). It has been proposed that the expression of CCL2 and CCR2 in astrocytes within acute and chronic lesions could play an important role in the chronic activation of immune cells (Tanuma et al., 2006). Our results suggest that older age at onset may be associated with increased CSF inflammation. A significant association with MCP-1/CCL2 and IL-8 was confirmed, also comparing patients with YORRMS and LORRMS. Notably, when analyzing patients with stable RR-MS, only MCP-1/CCL2 was significantly increased in the LORRMS group.

Finally, we found that, in patients with MS, LTP-like plasticity is reduced compared with healthy subjects and that older age was further negatively correlated with the LTP-like effect induced by iTBS, although also in healthy controls aging affects the expression of LTP-like plasticity as demonstrated by previous studies (Ghasemian-Shirvan et al., 2020). As efficient LTP-like plasticity expression has been previously associated with better clinical recovery after brain damage and with a clinically stable disease course in RR-MS (Stampanoni Bassi et al., 2019), the reduced LTP-like plasticity with aging could lessen the ability to compensate for ongoing brain damage, thereby promoting clinical progression (Mori et al., 2013). Notably, absent LTP-like plasticity has been proposed as a specific neurophysiological marker of progressive MS (Mori et al., 2013). Boosting synaptic plasticity with pharmacological treatments and the application of noninvasive brain stimulation, alone or combined with rehabilitation, could represent a potential therapeutic strategy to promote a stable disease course in these patients (Stampanoni Bassi et al., 2017; Nicoletti et al., 2020).

Taken together, our results show that older patients with MS present a peculiar endophenotype characterized by enhanced oxidative stress, increased central inflammation, and impaired synaptic plasticity, which may predispose them to PIRA. It has been demonstrated that PIRA plays a crucial role in disability worsening also in patients with RR-MS (San Francisco MS-EPIC Team et al., 2016, 2019; Tysabri® Observational Program (TOP) Investigators et al., 2018), challenging the traditional distinction between relapsing and remitting MS phenotypes (Kappos et al., 2020). It has been evidenced that ocrelizumab was more effective than IFN-β1a in preventing both disability accumulation related to relapses and PIRA in patients with RR-MS (Kappos et al., 2020). Prospective studies are needed to assess the impact of age and CSF inflammatory molecules on disability worsening and risk of disease progression.

## Conclusion

Our results suggest that patients with RR-MS with particular disease characteristics, such as increased CSF inflammatory biomarkers, reduced relapse activity, and increased oxidative stress, together with impaired expression of synaptic plasticity, may represent optimal candidates for recently approved therapies effective in preventing PIRA.

## Data Availability Statement

The raw data supporting the conclusions of this article will be made available by the authors, without undue reservation.

## Ethics Statement

The studies involving human participants were reviewed and approved by Ethics Committee of Neuromed hospital, Pozzilli (IS), Italy. The patients/participants provided their written informed consent to participate in this study.

## Author Contributions

MS, LG, EI, DC, and FB: study concept and design. LG, GG, RFa, and PB: acquisition of data. RFu, AF, MS, GM, and AMu: analysis of samples and data interpretation. AMo, TP, and JD: statistical analyses. MS, LG, and EI: participated in the drafting of the article. DC and FB: critical revision of the manuscript for important intellectual content. DC: study supervision. All authors contributed to the article and approved the submitted version.

## Funding

This study was supported by the FISM Grants (Fondazione Italiana Sclerosi Multipla-cod. 2019/S/1 to DC); the National Funding of the Italian Ministry of Universities and Research (MIUR-PRIN 2017-cod. 2017K55HLC to DC); the National Funding of the Italian Ministry of Health (RF-2018-12366144 to DC and GM; and Ricerca corrente to IRCCS San Raffaele Pisana, Ricerca corrente and 5 per mille public funding to IRCCS Neuromed).

## Conflict of Interest

RFu received honoraria for serving on scientific advisory boards or as a speaker from Biogen, Novartis, Roche, and Merck and funding for research from Merck. DC is an Advisory Board member of Almirall, Bayer Schering, Biogen, GW Pharmaceuticals, Merck Serono, Novartis, Roche, Sanofi-Genzyme, and Teva and received honoraria for speaking or consultation fees from Almirall, Bayer Schering, Biogen, GW Pharmaceuticals, Merck Serono, Novartis, Roche, Sanofi-Genzyme, and Teva. He is also the principal investigator in clinical trials for Bayer Schering, Biogen, Merck Serono, Mitsubishi, Novartis, Roche, Sanofi-Genzyme, and Teva. His preclinical and clinical research was supported by grants from Bayer Schering, Biogen Idec, Celgene, Merck Serono, Novartis, Roche, Sanofi-Genzyme and Teva. FB acted as Advisory Board members of Teva and Roche and received honoraria for speaking or consultation fees from Merck Serono, Teva, Biogen Idec, Sanofi, and Novartis and non-financial support from Merck Serono, Teva, Biogen Idec, and Sanofi. The remaining authors declare that the research was conducted in the absence of any commercial or financial relationships that could be construed as a potential conflict of interest.

## Publisher's Note

All claims expressed in this article are solely those of the authors and do not necessarily represent those of their affiliated organizations, or those of the publisher, the editors and the reviewers. Any product that may be evaluated in this article, or claim that may be made by its manufacturer, is not guaranteed or endorsed by the publisher.
