# Supplementary material for: Age at Disease Onset Associates With Oxidative Stress, Neuroinflammation, and Impaired Synaptic Plasticity in Relapsing-Remitting Multiple Sclerosis
Source: Front Aging Neurosci. 2021 Sep 10;13:694651. doi: 10.3389/fnagi.2021.694651 (PMC8461180; doi:10.3389/fnagi.2021.694651)
Supplement: Supplementary file 1 [file Table_1.docx]

|  | **Spearman’s ρ** | **p** | **B-H p** |
| --- | --- | --- | --- |
| **IL-1β** | -0.030 | 0.636 | 0.845 |
| **IL-2** | 0.015 | 0.819 | 0.948 |
| **IL-4** | 0.033 | 0.606 | 0.845 |
| **IL-5** | 0.073 | 0.252 | 0.792 |
| **IL-6** | 0.080 | 0.212 | 0.777 |
| **IL-7** | 0.067 | 0.298 | 0.792 |
| **IL-8** | **0.196** | **0.002** | **0.022** |
| **IL-9** | 0.039 | 0.542 | 0.845 |
| **IL-10** | 0.86 | 0.178 | 0.777 |
| **IL-12** | 0.111 | 0.082 | 0.451 |
| **IL-13** | 0.63 | 0.324 | 0.792 |
| **IL-15** | 0.033 | 0.609 | 0.845 |
| **IL-17** | 0.004 | 0.946 | 0.975 |
| **IL-1ra** | -0.029 | 0.646 | 0.845 |
| **G-CSF** | 0.029 | 0.649 | 0.845 |
| **GM-CSF** | 0.002 | 0.975 | 0.975 |
| **IP-10** | -0.015 | 0.811 | 0.948 |
| **IFNγ** | 0.035 | 0.589 | 0.845 |
| **TNF** | -0.029 | 0.653 | 0.845 |
| **MCP-1/CCL2** | **0.325** | **<0.001** | **<0.01** |
| **RANTES** | 0.009 | 0.888 | 0.975 |
| **MIP-1α/CCL3** | **0.177** | **0.005** | **0.037** |

**Supplementary table.** Correlations between Age at onset and CSF inflammatory molecules

Abbreviations: B-H, Benjamini-Hockberg; CSF, cerebrospinal fluid; IFN, interferon; IL, interleukin; MCP-1, monocyte chemoattractant protein 1; MIP-1α, macrophage inflammatory protein 1-alpha; TNF, tumor necrosis factor.
